# Seroprevalence of *Baylisascaris procyonis* Infection among Humans, Santa Barbara County, California, USA, 2014–2016

**DOI:** 10.3201/eid2308.170222

**Published:** 2017-08

**Authors:** Sara B. Weinstein, Camille M. Lake, Holly M. Chastain, David Fisk, Sukwan Handali, Philip L. Kahn, Susan P. Montgomery, Patricia P. Wilkins, Armand M. Kuris, Kevin D. Lafferty

**Affiliations:** University of California, Santa Barbara, California, USA (S.B. Weinstein, C.M. Lake, A.M. Kuris, K.D. Lafferty);; IRHC Inc., Atlanta Georgia, USA (H.M. Chastain);; Centers for Disease Control and Prevention, Atlanta, (H.M. Chastain, S. Handali, S.P. Montgomery, P.P. Wilkins);; Sansum Clinic, Santa Barbara (D. Fisk);; University of California, Berkeley, California, USA (P.L. Kahn);; US Geological Survey Western Ecological Research Center, Santa Barbara (K.D. Lafferty)

**Keywords:** nematode infections, enteric infections, parasites, Ascaridoidea, roundworm nematode, raccoon roundworm, zoonoses, raccoons, humans, California, prevalence, Baylisascaris procyonis

## Abstract

*Baylisascaris procyonis* (raccoon roundworm) infection is common in raccoons and can cause devastating pathology in other animals, including humans. Limited information is available on the frequency of asymptomatic human infection. We tested 150 adults from California, USA, for *B. procyonis* antibodies; 11 were seropositive, suggesting that subclinical infection does occur.

The raccoon roundworm (*Baylisascaris procyonis*) is a potential health risk to humans. Infected raccoons release eggs in their feces, and these eggs accumulate at communal defecation sites (latrines). When nonraccoon hosts consume eggs, larva migrans can cause blindness and fatal neurologic sequelae (*1*,*2*). Less than 5% of migrating larvae reach the brain, and experimental studies suggest that host size, infection site, and inoculating dose drive pathology (*1*).

Reported human disease cases are rare; however, there is growing evidence for more frequent asymptomatic infections. For example, a recent study showed that 7% of wildlife rehabilitators had *Baylisascaris*-specific antibodies (*3*). Large and heavily infected raccoon populations probably contaminate many regions with *B. procyonis* eggs (*1*). These microscopic eggs can survive for years (*4*), so anyone living in regions with infected raccoons probably has an exposure risk.

Santa Barbara County, California, USA, is a potential high-risk area for exposure to *B. procyonis* eggs. In Santa Barbara County, a baylisascariasis case was reported in a toddler in 2002 (*1*,*5*); raccoon roundworm consistently infects >80% of raccoons (*6*,*7*); and latrines are abundant in residential areas (J.F. Mendez, University of California, pers. comm., 2017 Feb 1), potentially exposing residents to roundworms. We describe the use of a parasite-specific antibody assay to detect subclinical *B. procyonis* infections in adult residents of Santa Barbara County. Recruitment, enrollment, and sampling methods were reviewed and approved by the Santa Barbara Cottage Hospital and University of California institutional review boards (14-06u).

## The Study

During 2014–2016, we provided public education about raccoon roundworm infection and offered free testing to healthy adults (18–75 years of age) who had lived in Santa Barbara County for >3 years. We recruited participants by word of mouth and flyers and through presentations at public outreach events and classes at the local university, natural history museum, zoo, and other venues. We also provided information about testing to local wildlife rehabilitators and researchers working with raccoons and *B. procyonis*. 

We collected serum samples from a convenience sample of 150 volunteers. This sampling included serum from 5 wildlife rehabilitators and 7 researchers; however, we considered results from these 12 persons separately because their *B. procyonis* roundworm exposure was expected to be higher than that for the general population.

We collected ≈5 mL of blood from each volunteer and allowed the samples to clot. We then centrifuged the samples at 1,500 × *g* for 15 min, separated them, and stored them at −80°C. Participants (149/150) filled out a questionnaire on demographic characteristics and potential risk factors, such as pet ownership, pet and wildlife feeding practices, past contact with raccoons or raccoon feces, and frequency of raccoon observations around their neighborhood and residence (Technical Appendix 1). We deidentified samples (Technical Appendix 2) and tested them for *B. procyonis* IgG by using a recombinant *B. procyonis* repeat antigen 1 protein Western blot assay, which has 88% sensitivity and 98% specificity (*3*,*8*). We generated prevalence estimates by using EpiTools (*9*), calculating 95% confidence limits (CLs) for an imperfect diagnostic assay (*10*). We then compared questionnaire responses from seropositive participants with those from seronegative participants by using exact binomial tests in R (*11*).

The 12 researchers and wildlife rehabilitators tested negative for *B. procyonis* antibodies. Among the remaining 138 volunteers, 11 tested positive (apparent prevalence 8.0%, Wilson CL 4.5%–13.7%; adjusted prevalence 6.9%, accounting for test sensitivity and specificity; Blaker exact CL 2.5%–13.4%). All 11 *B. procyonis*–seropositive participants had seen raccoons in their neighborhood during the past year, and 7 had seen 1 in their yard during the past month. Of the 11 *B. procyonis*–positive study participants, 9 reported no contact with raccoons or their feces; for the 2 who reported contact, the possible exposures occurred 2 and 12 months before testing. Persons with positive serologic test results ranged in age from 20 to 72 years and included an engineer, student, administrator, researcher, social worker, zoo volunteer, and retiree. Some seropositive persons owned dogs, fed animals outside, gardened, and had sandboxes. However, persons with negative serologic test results gave similar responses (p>0.15 for all comparisons), and we found too few infected persons to identify risk factors (Table).

**Table:** Questionnaire responses from participants in a study of the seroprevalence of *Baylisascaris procyonis* infection in humans, Santa Barbara County, California, USA, 2014–2016*

Variable	No. (%) seropositive respondents, n = 11	No. (%) seronegative respondents, n = 138
Sex		
M	3 (27)	54 (39)
F	8 (73)	84 (61)
Garden regularly	4 (36)	70 (51)
Sandbox at residence	1 (9)	11 (8)
Own a dog	4 (36)	37 (27)
Feed pets outside	1 (9)	11 (8)
Feed wildlife	1 (9)	18 (13)
Contact with raccoon or their feces	2 (18)	20 (14)
Raccoon seen in neighborhood in past		
Week	3 (27)	23 (17)
Month	4 (36)	45 (33)
Year	4 (36)	48 (35)
>1 y (or never)	0	22 (16)
Raccoon seen in yard in past		
Week	2 (18)	15 (11)
Month	5 (45)	33 (24)
Year	2 (18)	48 (35)
>1 y (or never)	2 (18)	42 (31)

*Responses are from 149/150 participants; 1 seronegative participant did not fill out the questionnaire.

## Conclusions

We estimate that ≈7% of the sampled persons had antibodies to raccoon roundworm; however, this convenience sample does not represent all county residents. Our recruiting strategy probably introduced income, age, and education biases, and, because we recruited study participants during presentations to groups interested in wildlife and outdoor activities, we might have selected a sample population with a greater exposure risk. Furthermore, because participants could receive their test results, we expect that persons concerned about past exposure were more likely to participate. Although most baylisascariasis is attributed to *B. procyonis*, cross-reactivity between the *B. procyonis* recombinant antigen assay and other less common *Baylisascaris* spp. is not well characterized and warrants further study. This survey suggests that subclinical *Baylisascaris* infection occurs in the general population; however, additional studies would improve prevalence estimates. These surveys could also include children because most clinical *B. procyonis* infections occur in persons <2 years of age (*1*,*2*) and it is unclear how long antibodies remain after exposure.

Despite frequent contact with raccoons and their feces, no sampled wildlife rehabilitators or researchers tested positive for *B. procyonis* antibodies. Most wildlife rehabilitators and all researchers examined were aware of *B. procyonis* and took precautions when handling raccoons, feces, and parasites. Although the finding of no infection in these high-risk groups could be a reflection of small sample size, it does suggest that preventive measures are effective.

Subclinical human *Baylisascaris* infections might occur wherever humans and infected raccoons overlap. These infections are likely more widespread than previously assumed, and their health risk remains an open question. Subclinical infection might result from lower intensity infection or depend on which tissues are infected (*1*). Low-intensity infection in organs, such as the brain, could result in subtle clinical manifestations, and clinical and serologic evidence is required to understand the full public health effect of *Baylisascaris* infections.

Technical Appendix 1 Questionnaire used in a study of the seroprevalence of *Baylisascaris procyonis* infection in humans, Santa Barbara County, California, USA, 2014–2016.

Technical Appendix 2 De-identified data collected in a study of the seroprevalence of *Baylisascaris procyonis* infection in humans, Santa Barbara County, California, USA, 2014–2016.
