# Supplementary material for: Seroprevalence of Baylisascaris procyonis Infection among Humans, Santa Barbara County, California, USA, 2014–2016
Source: Emerg Infect Dis. 2017 Aug;23(8):1397–9. doi: 10.3201/eid2308.170222 (PMC5547801; doi:10.3201/eid2308.170222)
Supplement: Technical Appendix 1 — Questionnaire used in a study of the seroprevalence of Baylisascaris procyonis infection in humans, Santa Barbara County, California, USA, 2014–2016. [file 17-0222-Techapp-s1.pdf]

Article DOI: https://doi.org/10.3201/eid2308.170222

Seroprevalence of *Baylisascaris procyonis* Infection among Humans, Santa Barbara County, California, USA, 2014–2016

Technical Appendix

Project Questionnaire

Participants filled out the project questionnaire in either a digital form on a tablet or a paper form. When participants used the tablet, address was indicated by placing a pin on a map. Full (de-identified) data are available in online Technical Appendix 2, https://wwwnc.cdc.gov/EID/article/23/8/17-0222-Techapp1.xlsx.

1. In what year were you born?

2. What is your race? (a. Asian or Pacific Islander, b. Black/African American, c. Hispanic/Latino, d. White/Caucasian, e. Other (please specify), f. Decline to state)

3. What is your sex? (a. Male, b. Female)

4. How many years have you lived in Santa Barbara County?

5. What is your occupation?

6. Do you do yard work or garden regularly? (a. Yes, b. No)

7. Do you have a play structure or sandbox at your residence? (a. Yes, b. No)

8. Does a dog live at your residence? (a. Yes, b. No)

9. Do you feed your pets outside? (a. Yes, b. No, c. No pets)

10. Do you feed wildlife at your residence? (a. Yes, b. No)

11. Have you ever been in physical contact with a raccoon or raccoon feces? (a. Yes, b. No)

a. How long ago did this occur?

12. How long has it been since you saw a raccoon in your neighborhood? (a. In the last week, b. In the last month, c. In the last year, d. Never)

13. How long has it been since you saw a raccoon around your house or yard? (a. In the last week, b. In the last month, c. In the last year, d. Never)

14. What is your street address?
